# A novel use of hill function and utility of ^99m^Tc-MIBI scintigraphy to detect earlier lower extremity microvascular perfusion in patients with type 2 diabetes

**DOI:** 10.1097/MD.0000000000008038

**Published:** 2017-09-22

**Authors:** Yingsha Li, Qiang Li, Shenju Liang, Xia Liang, Wen Zhou, Hongbo He, Rongbing Jin, Kaifa Wang, Zhiming Zhu, Zhencheng Yan

**Affiliations:** a Center for Hypertension and Metabolic Diseases, Department of Hypertension and Endocrinology; b Department of Nuclear Medicine, Daping Hospital; c School of Biomedical Engineering, Third Military Medical University, Chongqing, P.R. China.

**Keywords:** ^99m^Tc-MIBI scintigraphy, lower extremity microvascular perfusion, type 2 diabetes

## Abstract

We use the Hill function to analyze the dynamics of Tc-99m 2 methoxy-isobutyl-isonitrile (^99m^Tc-MIBI) scintigraphy data and to examine the earlier lower extremity microvascular perfusion of diabetic patients without typical clinical symptoms and with the preserved normal ankle-brachial index (ABI).

Eighty-eight participants (30 healthy control, 34 diabetic patients, and 24 diabetic patients with peripheral arterial disease [PAD]) were recruited and applied ^99m^Tc-MIBI scintigraphy. Fourteen diabetic patients with PAD also underwent computed tomography angiography (CTA) examination and were performed endovascular interventions.

Diabetic patients with normal ABI already have significantly impaired maximum ^99m^Tc-MIBI muscle perfusion counts (*P* < .001) and the peak times of the lower extremity muscle perfusion (*P* < .05). ^99m^Tc-MIBI scintigraphy showed great consistent with ABI and CTA in detecting PAD. ^99m^Tc-MIBI scintigraphy was also found to be effective in evaluating lower extremity circulation after endovascular interventions (*P* < .05).

Hill function-based analysis of ^99m^Tc-MIBI scintigraphy might be effective method to evaluate earlier lower extremity perfusion changes in diabetic patients.

## 1 Introduction

Peripheral arterial disease (PAD) is an atherosclerotic disease frequently accompanied with diabetes which severely worsens the lower extremity function.^[1]^ The early diagnosis and evaluation of lower extremity microvascular perfusion in diabetic patients are very important for prompt treatment.

Impairment of microcirculation represented by lower extremity muscle perfusion has already occurred in patients without typical clinical symptoms.^[2]^ However, current clinic indices for the evaluation and detection of PAD are usually focused on large vessels. Nuclear imaging approaches have superior in the assessment of muscle perfusion and have been used in quantitatively assessment of lower extremity perfusion in patients.^[3–6]^ However, its clinical application is limited by the lacking of standard analysis method. Hill function is a concentration-response function, which can be candidate for simulating tissue and cellular uptake of Tc-99m 2 methoxy-isobutyl-isonitrile (^99m^Tc-MIBI) dynamics. This study took a novel Hill function-based analysis of ^99m^Tc-MIBI scintigraphy data, to evaluate earlier lower extremity perfusion changes in diabetic patients.

## 2 Materials and methods

### 2.1 Study design, imaging acquisition, and analysis

**Figure 1:**
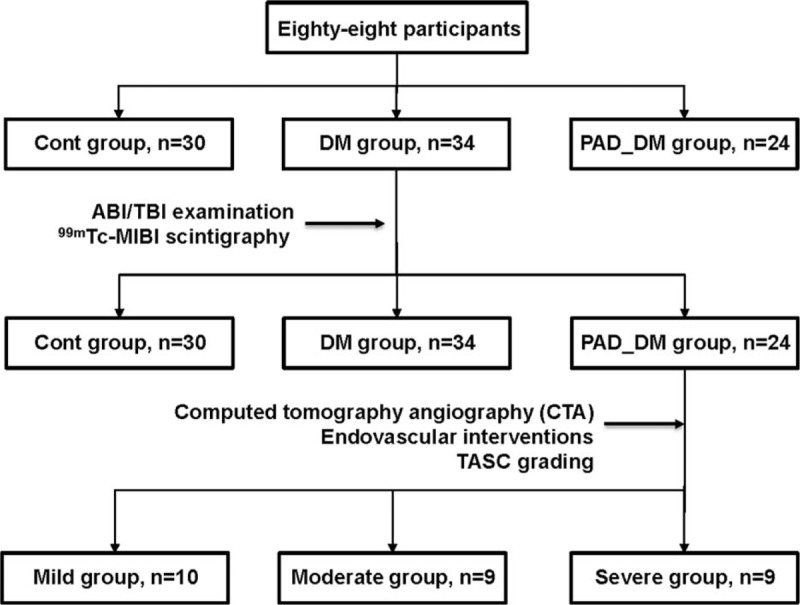
Flow chart representing the participants’ selection and breakdown of the group intervention. Note that the sample size in TASC grading-based groups was the number of limbs. TASC = trans-atlantic inter-society consensus document.

Eighty-eight participants were recruited and assigned to the control group (Cont, had no history of diabetes or PAD, n = 30), diabetes group (DM, with normal ankle-brachial index (ABI), n = 34), and diabetes with the PAD group [diabetes with the PAD group (PAD_DM), n = 24]. ABI is obtained by using the blood pressure differential between the brachial systolic blood pressure and systolic blood pressure from the posterior tibial artery, with a hand-held Doppler (Huntleigh, Cardiff, CF24 5HN, UK). For the lower extremity muscle perfusion assessment, the intravenous injection of a 370-640 MBq (10-20 mCi) bolus of ^99m^Tc-MIBI (College of Chemistry, Beijing Normal University, China) were performed, and serial dynamic posterior planar images were recorded for 5 minutes in the regions of both calves. Using an image analysis device (Xeleris 3, GE Medical System), serial summed images and time-activity curves were created from the data. Fourteen patients from the PAD_DM group who underwent CTA and performed endovascular interventions were further divided into 3 groups according to the trans-atlantic inter-society consensus document (TASC) grading method,^[7]^ namely a mild group (TASC A), a moderate group (TASC B), and a severe group (TASC C and D). A flow chart representing the above participants’ selection and breakdown of the group intervention was provided (Fig. 1). Three independent investigators evaluated the images while blinded to the participants’ clinical and ultrasound data. This study was approved by the hospital ethical committee and written informed consent was obtained from all the participants.

Originally, the Hill function was proposed to describe the in vivo concentration response relationship,^[8]^ which fits concentration-response curve data. Similarly, the tissue uptake of ^99m^Tc-MIBI is proportional to blood flow, and its cellular uptake and retention are dependent on mitochondrial and plasma membrane potentials.^[9]^ Hill function parameters were fitted using the greedy algorithm method implemented in Matlab software (Mathworks). Since the matching of target distribution is associated with simulating steps, we used 100,000 steps to ensure the accuracy of our parameter distributions.

The Hill function can be written as 
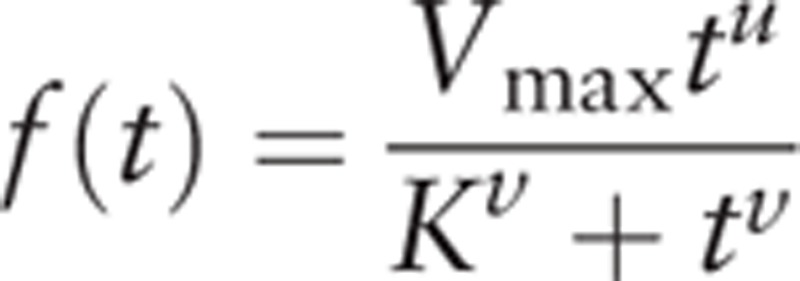


*V*_max_: the maximum ^99m^Tc-MIBI perfusion counts in the tissue.

*t*: time-dependent observed ^99m^Tc-MIBI perfusion counts in the tissue.

*K*: under the condition of *u* = *v*, half-saturation constant ^99m^Tc-MIBI perfusion counts.

*u*, *v*: constants of effect response, and their ratio represents the steepness of the effect-concentration curve, which is a measure of how strongly the ^99m^Tc-MIBI perfusion counts changes in response to changes in time.

Let 
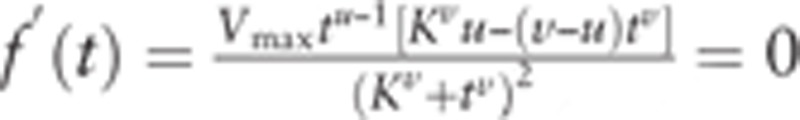
. We can derive the peak time of the curve, 
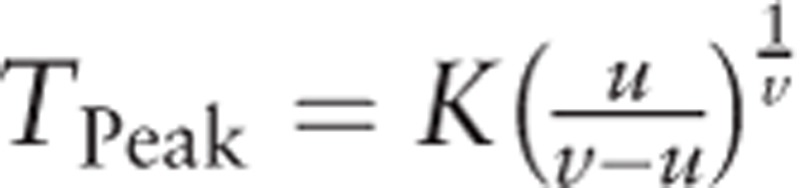
, which is the measurement of muscle perfusion ability.

### 2.2 Statistics analysis

All results were presented as mean ± SD. Differences among groups were compared by means of the chi-squared test for categorical variables, and 1-way analysis of variance (ANOVA) with Bonferroni's multiple comparison post-hoc test and Students’ *t*-test for continuous variables of 3 or 2 groups comparison respectively. A 2-sided *P* value of less than 0.05 was considered to indicate statistical significance. All statistical analyses were conducted with the use of SPSS software, version 13.0 (SPSS Inc.).

## 3 Results

### 3.1 Baseline characteristics of the participants

The clinical baseline characteristics of the participants in each group are presented in Table 1. No differences were found in age, gender, serum total cholesterol (TC), triglyceride (TG), and High density lipoprotein cholesterol among groups. Although being apparently distinct from the Cont group, no differences were found in HbA1c, fasting blood glucose, high sensitivity-C-reactive protein (*hs*-CRP), and low density lipoprotein cholesterol between DM and PAD_DM groups. The diabetic patients with PAD exhibited profoundly decreased ABI values (*P* < .001) compared with the other groups.

**Table 1:**
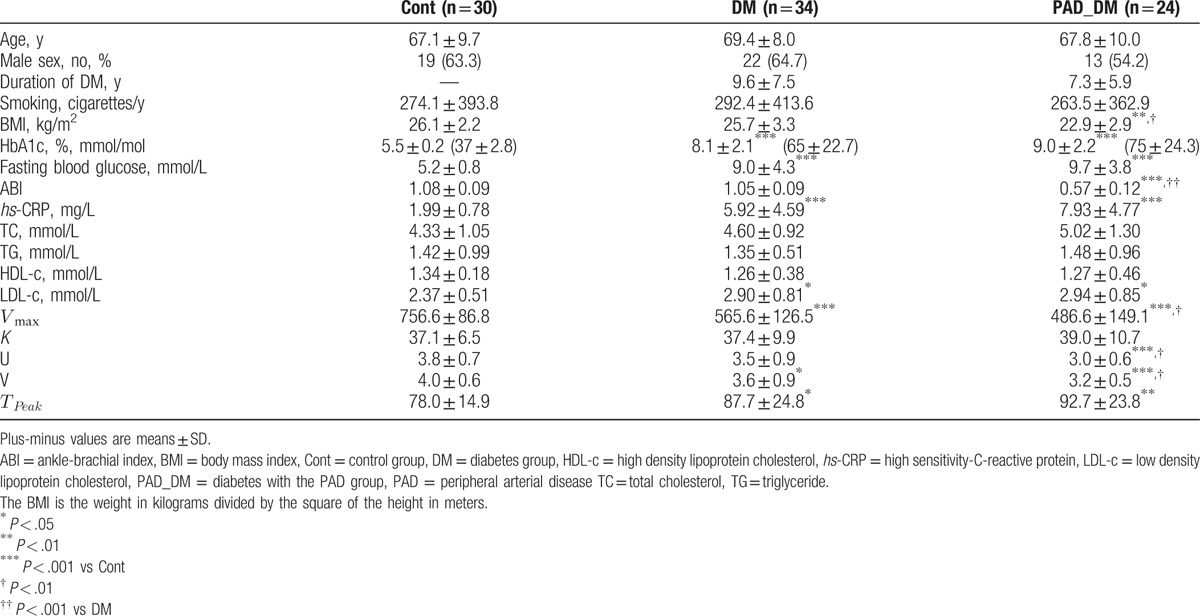
Baseline characteristics of the participants.

### 3.2 Earlier changes in lower extremity muscle perfusion in the diabetic patients

The estimated Hill function parameters using the ^99m^Tc-MIBI muscle perfusion data are also provided in Table 1. However, with normal ABI values, diabetic patients already had significantly decreased maximum ^99m^Tc-MIBI muscle perfusion counts compared with healthy participants (565.6 ± 126.5 vs 756.6 ± 86.8, *P* < .001), and decreased lower extremity muscle perfusion became more profoundly in the DM_PAD group. No differences were found in half saturated ^99m^Tc-MIBI muscle perfusion counts among 3 groups, which may represent the smaller differences of the basic characteristics among groups. The peak times of the lower extremity muscle perfusion of DM and DM_PAD groups were significantly prolonged compared with the Cont group (87.7 ± 24.8 vs 78.0 ± 14.9, *P* < .05; 92.7 ± 23.8 vs 78.0 ± 14.9, *P* < .01), whereas the results showed no significant relation with PAD.

### 3.3 Comparison of ^99m^Tc-MIBI scintigraphy with CTA and ABI in the evaluation of lower extremity perfusion

**Figure 2:**
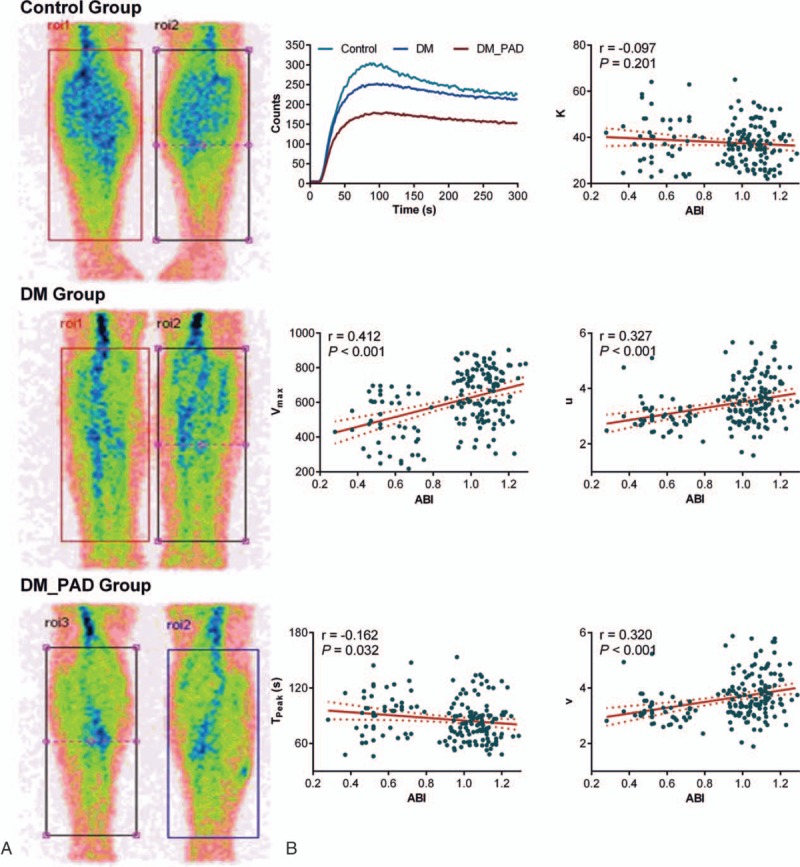
(A) Representative posterior planar images following the injection of ^99m^Tc-MIBI marked on both calves in the cases of each group. (B) Correlations of the ^99m^Tc-MIBI scintigraphy perfusion parameters with the individual ABIs. Linear regression was used for the analyses. The solid line corresponds to the regression line, and the dashed line represents the 95% confidence band of the best-fit line. ^99m^Tc-MIBI = Tc-99m 2 methoxy-isobutyl-isonitrile, ABI = ankle-brachial index.

**Figure 3:**
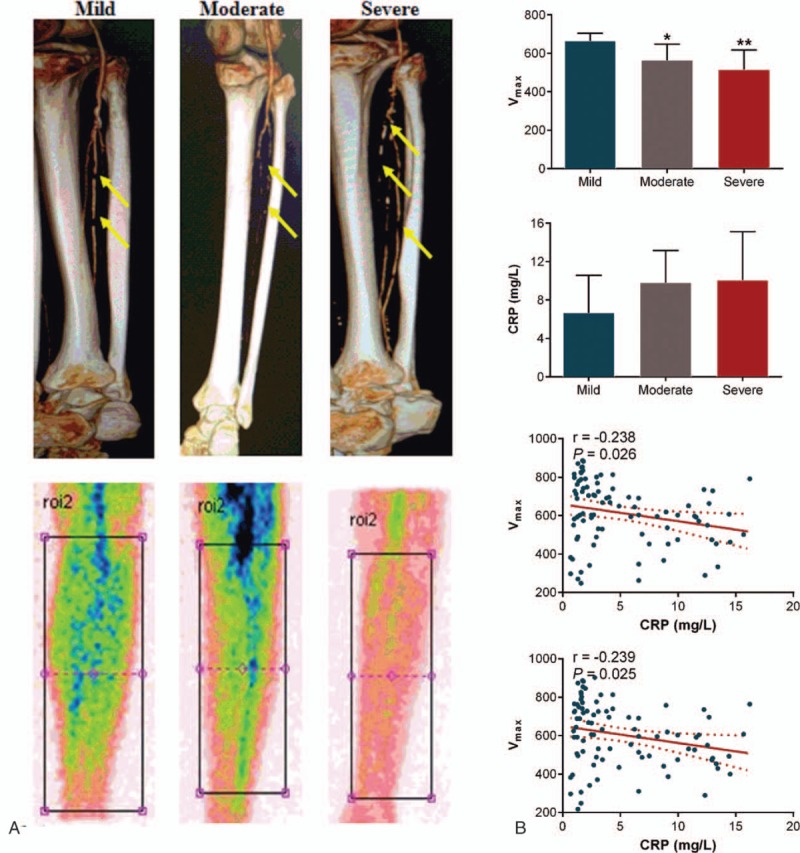
(A) Representative CTA and ^99m^Tc-MIBI scintigraphy images obtained from 3 patients according to the TASC grade. Lower limb arteriosclerosis is indicated by the arrows. (B) The numerical analyses of the muscle perfusion parameters and *hs-*CRP according to the TASC vascular grade following the CTA examinations. ^∗^*P* < .05, ^∗∗^*P* < .01 vs mild. Linear regression was used for analyzing correlations of the muscle perfusion parameters of both legs with the individual *hs-*CRP. The solid line corresponds to the regression line, and the dashed line represents the 95% confidence band of the best-fit line. ^99m^Tc-MIBI = Tc-99m 2 methoxy-isobutyl-isonitrile, CTA = computed tomography angiography, *hs*-CRP = high sensitivity-C-reactive protein.

To examine the sensitivity of Hill function parameters in detecting PAD occurrence, strong consistencies of ABI and the Hill function parameters regarding lower limb microvascular perfusion were observed (Fig. 2). Based on the CTA results and TASC vascular grades, 14 patients who underwent CTA examinations were further divided into 3 groups (i.e., mild, moderate, and severe, 10 limbs in 8 cases, 9 limbs in 7 cases, and 9 limbs in 8 cases, respectively). The maximum ^99m^Tc-MIBI muscle perfusion counts decreased dramatically with the severity of the vascular lesions represented by the TASC vascular grade increased (Fig. 3). Although no differences of *hs*-CRP were observed among groups, the maximum ^99m^Tc-MIBI muscle perfusion counts showed significantly inverse correlation with the *hs*-CRP level (Fig. 3).

### 3.4 Assessment of the efficiency of endovascular interventions in the lower extremities using ^99m^Tc-MIBI scintigraphy

**Figure 4:**
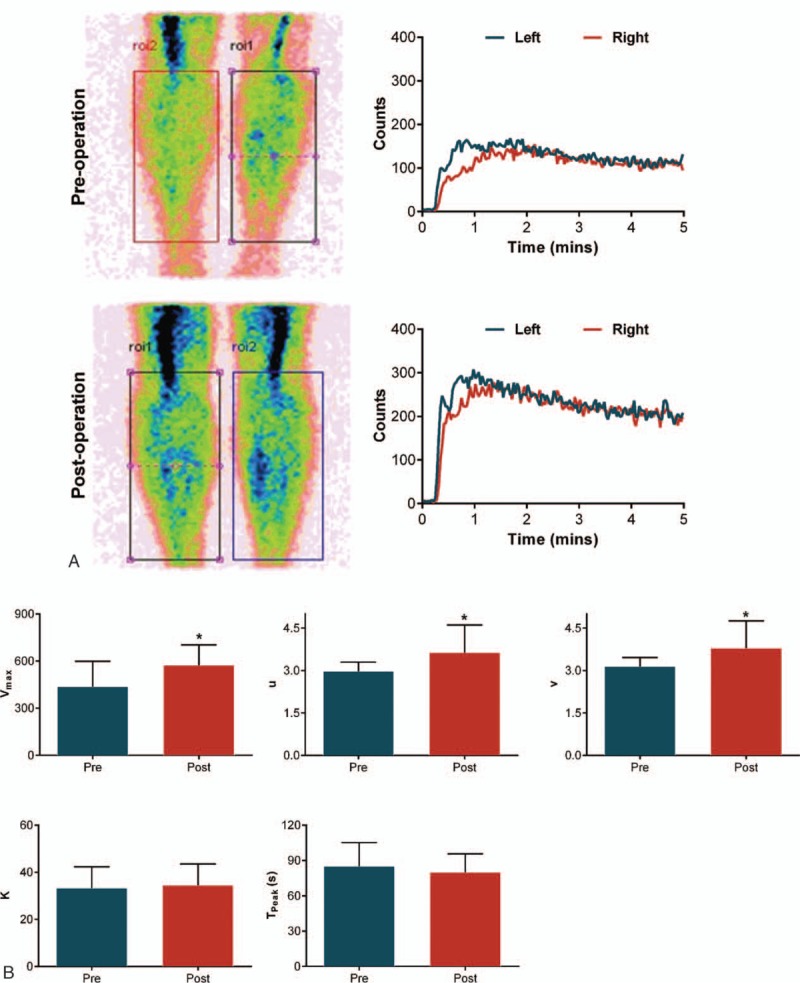
(A) Posterior planar images and the time-activity curves at the 5th minute following the injection of ^99m^Tc-MIBI in the ROIs marked on both calves pre- and postoperation. (B) Improved muscle perfusion parameters of calves following vascular surgery. ^∗^*P* < .05 vs preoperation. ^99m^Tc-MIBI = Tc-99m 2 methoxy-isobutyl-isonitrile, ROIs = region of interests.

Potential application of ^99m^Tc-MIBI scintigraphy in assessing the efficiency of improvements in lower extremity microcirculation in the diabetic patients who underwent endovascular interventions was also examined. Apparent improvements in muscle perfusion parameters were observed in calves (*P* < .05), although the peak time of the muscle perfusion failed to reach statistical significance (Fig. 4).

## 4 Discussion

This novel study is the first to describe the use of Hill function in analyzing ^99m^Tc-MIBI scintigraphy data to evaluate earlier lower extremity microvascular perfusion in diabetic patients. We found that asymptomatic diabetic patients with normal ABI already have significantly impaired lower extremity muscle perfusion. ^99m^Tc-MIBI scintigraphy showed great consistent with ABI and CTA in detecting PAD, whereas exhibited advantages in evaluating lower extremity microcirculation. ^99m^Tc-MIBI scintigraphy was also found to be effective in assessing lower extremity microcirculation after endovascular interventions.

Hemodynamic changes in the lower extremities frequently occur before the onset of PAD and even before the diagnosis of diabetes, measurements of muscular microcirculation could be of considerable importance in the diagnosis of earlier lower extremity microvascular perfusion. Techniques for restenosis detection include ABI measurements, duplex ultrasound, MR imaging, and CT angiography. These methods have some shortcomings in the evaluation of the degree and extent of tissue blood perfusion, especially in the early stage of diabetes.^[10]^^99m^Tc-MIBI scintigraphy has been used to assess lower extremity perfusion in PAD and diabetic patients, which showed high sensitivity and specificity.^[2–6]^ However, several major limitations restrict its clinic application.

The rest and stress perfusion were usually used as the indication of earlier impaired microcirculation. However, there is no unified operational protocol and methods of stress test are diverse. Second, the total counts of the selected regions were used in previous studies, which ignoring the hemodynamic changes of muscle perfusion. Finally, previous statistic analysis needed to select reference ^99m^Tc-MIBI uptake as control, and variate standards used in different studies lead to poor comparability. We used the Hill function to simulate time-activity data of ^99m^Tc-MIBI scintigraphy, which largely minimized artificial and bias of statistical methods. In accordance with the biological meanings of the Hill function parameters, we found significant reduction in the maximum ^99m^Tc-MIBI perfusion counts and the peak time of the curve in asymptomatic diabetic patients with normal ABI.

This study has several limitations. First, this is an observational study, and the generality of its results to other countries is still unclear. Second, our study did not include nondiabetic patients with PAD, and the difference between them and diabetic patients with PAD needs further study. Third, due to the lack of well-documented statistical methods on analyzing ^99m^Tc-MIBI perfusion data, we cannot draw receiver operating characteristic (ROC) curve to evaluate our method's diagnostic accuracy.

This study provided an effective and noninvasive method for the earlier evaluation of lower limb microvascular perfusion in diabetic patients via the use of Hill function based ^99m^Tc-MIBI scintigraphy analysis. Further studies of the indications for ^99m^Tc-MIBI scintigraphy in diabetic patients are warranted.

## Acknowledgments

The authors thank Dan Wang from Department of Nuclear Medicine for technical assistance. They also thank all the patients and investigators for their participation.
